# Proteolysis in Reproduction: Lessons From Gene-Modified Organism Studies

**DOI:** 10.3389/fendo.2022.876370

**Published:** 2022-05-04

**Authors:** Daiji Kiyozumi, Masahito Ikawa

**Affiliations:** ^1^ Research Institute for Microbial Diseases, Osaka University, Suita, Japan; ^2^ PRESTO, Japan Science and Technology Agency, Kawaguchi, Japan; ^3^ The Institute of Medical Science, The University of Tokyo, Tokyo, Japan; ^4^ CREST, Japan Science and Technology Agency, Kawaguchi, Japan

**Keywords:** protease, fertilization, proteolysis, protease inhibitor, pseudoprotease, gene-modified animal models, ubiquitin-proteasome system, sperm maturation

## Abstract

The physiological roles of proteolysis are not limited to degrading unnecessary proteins. Proteolysis plays pivotal roles in various biological processes through cleaving peptide bonds to activate and inactivate proteins including enzymes, transcription factors, and receptors. As a wide range of cellular processes is regulated by proteolysis, abnormalities or dysregulation of such proteolytic processes therefore often cause diseases. Recent genetic studies have clarified the inclusion of proteases and protease inhibitors in various reproductive processes such as development of gonads, generation and activation of gametes, and physical interaction between gametes in various species including yeast, animals, and plants. Such studies not only clarify proteolysis-related factors but the biological processes regulated by proteolysis for successful reproduction. Here the physiological roles of proteases and proteolysis in reproduction will be reviewed based on findings using gene-modified organisms.

## Introduction

Although a simple peptide bond between two amino acids in water at room temperature has a half-life of several years (1), the hydrolysis of a peptide bond is significantly accelerated under the presence of proteases. As well as mediating non-specific protein hydrolysis, proteases also act as processing enzymes that perform highly selective, limited, and efficient cleavage of specific substrates. As many biological processes are influenced by this irreversible post-translational protein modification, dysregulation of the expression and/or function of proteases underlie many human pathological processes and have therefore been an intensely studied class of targets for drug discovery.

By searching *Saccharomyces cerevisiae*, *Drosophila melanogaster*, and *Caenorhabditis elegans* genome databases with a gene ontology term “peptidase activity” (GO:0008233), 51, 506, and 448 genes encoding proteases, respectively, can be identified (2–4). In the mouse and human genome, 628 and 553 protease genes exist, respectively (5). In *Arabidopsis thaliana*, 723 protease genes were reported (6). Based on catalytic mechanisms, proteases can be divided into five classes: cysteine proteases, serine proteases, metalloproteases, threonine proteases, and aspartic proteases. After activation of the amide, cysteine, serine, and threonine proteases utilize the namesake residue to attack the amide carbonyl group, whereas metalloproteases and aspartic proteases use an activated water molecule as a nucleophile. As proteases bind their substrates between the substrate side chains and well-defined substrate-binding pockets within the active site, they have their own preference for substrate amino acid sequence proximal to the cleavage site (7). There are some enzymatically inactive pseudoproteases encoded in the mammalian genome in which the amino acid residues indispensable for catalytic activity are substituted. As proteases are potentially toxic, their activities are strictly regulated as such by pH, specific ion concentrations, posttranslational modifications, and spatiotemporal expression of protease inhibitors.

The contribution of proteases depends on their intracellular or extracellular localization where they act on substrate proteins. The ubiquitin-proteasome system (UPS) is a complex but sophisticated intracellular proteolytic system in eukaryotes; this complex system degrades unneeded or damaged proteins by proteolysis. When target proteins are post-translationally labeled with ubiquitin, a protein of 76-amino acid residues exhibiting high sequence conservation among eukaryotes, they will be recognized and degraded by the proteasome.

Proteolytic processing events are fundamental in reproductive processes including gametogenesis, fertilization, and embryonic development. Recent advances in generating gene-modified animals have identified many proteases and their regulators associated with reproduction in various species including yeast, invertebrates, vertebrates, and plants. In the following sections the physiological importance of proteolysis in reproduction will be overviewed based on findings obtained by gene-modified organism studies. Proteolysis-related genes essential in reproduction identified by gene-modified animal studies are listed in **Table 1**. Few proteins are known to be proteolytically processed under certain reproductive situations. They are, however, not included in this review as the physiological roles of such processing in reproduction are not fully clarified at present.

**Table 1:** Proteolysis-related genes associated with reproduction.

Gene	Protein feature	Protein localization	Gene-modified organism	Fertility	Phenotype	Refs.
***S. cerevisiae***						
*ste24*	Prenyl protein-specific endoprotease	Intracellular membrane	Ethylmethane-sulfonate (EMS) mutagenesis	Sterile	MAT a-specific sterility.	(8)
*axl1*	Metalloprotease	Intracellular	UV exposure	Sterile	Defect in a-factor pheromone secretion.	(9)
***C. elegans***						
*cpi-2a*	Cystatin-like cysteine protease inhibitor	Extracellular	Deletion mutant	Sterile	Oocyte-specific sterility.	(10)
*gon-1*	Metalloprotease	Extracellular	EMS mutagenesis	Sterile	Gonadal developmental defect.	(11, 12)
*timp-1*	Metalloprotease inhibitor	Extracellular	Trimethylpsoralen (TMP)–UV-mutagenesis	Sterile	Gonadal growth defect.	(13)
*dss-1*	26S proteasome subunit	Intracellular	Deletion mutant	Sterile	Defects in oogenesis.	(14)
*dpf-3*	Serine protease	Intracellular	Deletion mutant	Sterile	Impaired spermatogenesis.	(15)
*sup-17*; *adm-4*	ADAM metalloproteases	Cell membrane	EMS mutagenesis; TMP)–UV-mutagenesis	Sterile	Aberrant spermathecal function.	(16)
*pam-1*	Metalloprotease	Intracellular	RNAi knockdown	Subfertility	Decreased brood size. Expanded pachytene.	(17)
*try-5*	Serine protease	Extracellular	Deletion mutant	Fertile	*try-5* functions in parallel to *spe-8* for male fertility.	(18)
*swm-1*	Trypsin inhibitor-like	Extracellular	EMS mutagenesis	Reduced male fertility	Ectopic sperm activation within the male reproductive tract. Failure of sperm transfer to hermaphrodite.	(19)
*gcna-1*	Metalloprotease	Nucleus	Deletion by CRISPR/Cas9	Fertility defects	Decrease of fertility in later generations because of genomic instability	(20)
*T12E12.6*	Metalloprotease	Intracellular	RNAi knockdown	Subfertility	Decreased brood size.	(17)
*zmp-2*	Metalloprotease	Extracellular	RNAi knockdown	Subfertility	Reduced offspring production.	(21)
***D. melanogaster***						
*CG9000*; *CG9001*; *CG9002*	Yeast ste24p ortholog proteases	Intracellular membrane	Ends-out gene targeting	Male fertility defects	Abnormal spermatid maturation.	(22)
*Prosalpha6T*	Proteasome subunit	Intracellular	KO	Male infertility	Spermatogenic defects in sperm individualization and nuclear maturation.	(23)
*Duba*	Deubiquitylating enzyme	Intracellular	Imprecise P-element excision	Male infertility	Defects in spermatid individualization.	(24)
*Dronc*	Cysteine protease	Intracellular	Transgenic expression of dominant-negative DRONC	Uncertain	Defects in spermatid individualization.	(25)
*Dredd*	Cysteine protease	Intracellular	EMS mutagenesis	Fertile	Defects in spermatid individualization.	(25)
*Dark*	Caspase activator	Intracellular	Enhancer trap	Male infertility	Defects in spermatid individualization.	(25)
*Htra2*	Serine protease	Mitochondria	P element mobilization	Male infertility	Sperm were completely immotile	(26)
			EMS mutagenesis	Male infertility	Defective spermatogenesis.	(27)
*S-Lap1-8*	Leucylamino-peptidase	Intracellular	Classical mutant, CRISPR/Cas9	Male infertility or subfertility	Deficient accumulation of paracrystalline material in mitochondria.	(28)
*Sems*	Trypsin-like protease	Extracellular	Knockdown	Male subfertility	Females laid fewer number of eggs when mated to *Sems* knockdown males. Sperm remained in storage in the seminal receptacle.	(29)
*Nep4*	Metalloprotease *Mmel1* ortholog	Cell membrane	KO	Male infertility	Mutant sperm are quickly discarded by females.	(30)
*Dcp-1*	Cysteine protease	Intracellular	Female carrying germline KO clone	Female infertility	Defective oogenesis.	(31)
*mh*	Metalloprotease	Nucleus	EMS mutagenesis, P element mobilization	Female infertility	The integration of paternal chromosomes in the zygote was specifically affected.	(32, 33)
*Ance*	Angiotensin-converting enzyme	Extracellular	EMS mutagenesis	Male infertility	Compound heterozygotes for two different lethal alleles are male sterile.	(34)
*Slfc*	Serine protease	Extracellular	RNAi knockdown	Male infertility	Details are unknown. Females also show slightly decreased fertility.	(35)
*ome*	Serine protease	Cell membrane	EMS mutagenesis	Male subfertility	Details are uncertain.	(36)
*Mmp2*	Metalloprotease	Extracellular	RNAi	Female subfertility	Ovulation was blocked.	(37)
***A. socius***						
*ejac-sp*	Serine protease	Extracellular	RNAi knockdown	Male subfertility	Reduced ability to induce a female to lay eggs.	(38)
***Bombyx mori***						
*Osp*	Serine protease	Cell membrane	KO	Female infertility	Mutant females laid fewer eggs than wild-type females and eggs did not hatch	(39)
*Ser2*	Serine protease	Extracellular	KO	Male infertility	Wild-type females mated with mutant males laid eggs normally but the eggs did not hatch.	(40)
***Spodoptera litura***						
*Osp*	Serine protease	Cell membrane	KO	Female infertility	Mutant females laid fewer eggs than wild-type females and eggs did not hatch.	(39)
***Plutella xylostella***						
*Ser2*	Serine protease	Extracellular	KO	Male infertility	Mutant sperm morphology is normal but they do not enter eggs.	(40)
***Hyphantria cunea***						
*Hcser2*	Serine protease	Extracellular	RNAi knockdown, KO	Male infertility	The growth, development, mating behavior, or egg laying was not affected.	(41)
***Bactrocera dorsalis***						
*Bdcp-1*	Cysteine protease	Intracellular	RNAi knockdown	Female infertility	Impaired ovary development.	(42)
***M. musculus***						
*Psma8*	Proteasome component	Nucleus	KO	Male infertility	Arrested spermatogenesis at spermatocyte stage.	(43)
*Psme3*	Proteasome activator	Intracellular	KO	Male subfertility	Decreased sperm number and motility.	(44)
*Psme4*	Proteasome activator	Nucleus	KO	Severe male subfertility	Defective spermatogenesis.	(45)
*Psme3*; *Psme4*	Proteasome activator	Intracellular	Double KO	Male infertility	Morphologically normal sperm with motility defect.	(46)
*Cops5*	Metalloprotease	Intracellular	KO	Male infertility	Male infertility. Germ cells undergo significant apoptosis at a premeiotic stage.	(47)
*Usp2*	Ubiquitin-specific protease	Nucleus	KO	Male subfertility	Defects in sperm motility.	(48)
*Usp9x*	Ubiquitin-specific protease	cytoplasm	*Vasa-cre*; *Usp9x* ^fl/Y^	Male infertility	Apoptosis of spermatocytes.	(49)
*Usp26*	Ubiquitin-specific protease	Intracellular	KO	Severe male subfertility	Unsynapsed chromosomes in pachynema and defective chiasma formation in diplonema, apoptosis of metaphase spermatocytes and decrease of spermatids.	(50, 51)
*Usp1*	Ubiquitin-specific protease	Nucleus	KO	Male infertility	Impaired spermatogenesis.	(52)
*Apaf1*	Caspase activator	Intracellular	KO	Male infertility	Degeneration of spermatogonia resulting in the absence of sperm.	(53)
*Agbl5*	Metalloprotease	Intracellular	KO	Male infertility	Defective spermatogenesis	(54, 55)
*Gcna*	Metalloprotease	Nucleus	KO	Male infertility	Nearly devoid of sperm.	(56)
*Tasp1*	Endopeptidase	Nucleus	KO	Male infertility	Release immature germ cells.	(57)
*Tysnd1*	Serine protease	Peroxisome	KO	Male infertility	Globozoospermia, no acrosomal cap.	(58)
*Spink2*	Serine protease inhibitor	Extracellular	KO	Male infertility	Oligoasthenoteratozoospermia in heterozygotes, azoospermia in homozygotes.	(59)
*Serpina5*	Serine protease inhibitor	Extracellular	KO	Male infertility	Abnormal spermatogenesis due to destruction of the Sertoli cell barrier.	(60)
*Adamts2*	Metalloproteinase	Extracellular	KO	Male infertility	Marked decrease in testicular sperm.	(61)
*Acr*	Serine protease	Acrosome	KO	Male subfertility	Delayed fertilization.	(62, 63)
*Pcsk4*	Serine protease	Acrosomal membrane	KO	Male subfertility	Putatively due to impaired fertilization.	(64, 65)
*Tmprss12*	Serine protease	Plasma membrane	KO	Male infertility	Deficient sperm migration into oviduct.	(66)
*Prss55*	Serine protease	Plasma membrane	KO	Male infertility	Deficient sperm migration into oviduct.	(67, 68)
*Tryx5*	Serine protease	Plasma membrane	KO	Male infertility	Deficient sperm migration into oviduct.	(69)
*Prss37*	Pseudoprotease	Plasma membrane	KO	Male infertility	Deficient sperm migration into oviduct.	(70)
*Ace*	Metallo-carboxypeptidase	Plasma membrane	KO	Male subfertility	Deficient sperm migration into oviduct.	(71)
*Adam1a*	Pseudoprotease	Plasma membrane	KO	Male infertility	Deficient sperm migration into oviduct.	(72)
*Adam2*	Pseudoprotease	Plasma membrane	KO	Male subfertility	Deficient sperm migration into oviduct	(73)
*Adam3*	Pseudoprotease	Plasma membrane	KO	Male infertility	Deficient sperm migration into oviduct.	(74, 75)
*Adam6*	Pseudoprotease	Plasma membrane	KO	Male infertility	Deficient sperm migration into oviduct.	(76)
*Cst8*; *Cst9*; *Cst11*; *Cst12*; *Cst13*; *Cstdc1*; *Cstdc2*; *Cstl1*	Cystatin-like inhibitor	Extracellular	Multiple KO	Male infertility	Deficient sperm migration into oviduct.	(77)
*Ovch2*	Serine protease	Extracellular	KO	Male infertility	Deficient sperm migration into oviduct.	(78)
*Mmel1*	Metalloprotease	Extracellular	KO	Male infertility	Normal spermatogenesis but reduced egg fertilization.	(79)
*Prss21*	Serine protease	Plasma membrane	KO	Male subfertility, decreased *in vitro* fertility	Mutant spermatozoa possessed decreased motility, angulated and curled tails, and fragile necks. Decreased *in vitro* zona pellucida binding and acrosome reaction.	(80, 81)
*Cpe*	Metalloprotease	Extracellular	Spontaneous mutation	Male subfertility	Abnormal sexual behavior. Abnormal testis morphology in older mutant males.	(82)
*Adam24*	Pseudoprotease	Plasma membrane	KO	Male subfertility	Polyspermic fertilization.	(83)
*Adam7*	Pseudoprotease	Plasma membrane	KO	Male subfertility	Decreased cell height in caput epididymis, spermatic granuloma, kinked sperm flagellum and reduced sperm motility.	(84)
*Cst3*	Cysteine protease inhibitor	Extracellular	KI (Leu68Gln)	Male subfertility	Reduced viability of spermatozoa and large agglutinated clumps.	(85)
*Serpine2*	Serine protease inhibitor	Extracellular	KO	Male subfertility	Inadequate semen coagulation and deficient vaginal plug formation upon copulation	(86)
*Tmprss6*	Serine protease	Plasma membrane	KO	Female infertility	Marked retardation in ovarian maturation.	(87)
*Ambp*	Serine protease inhibitor	Extracellular	KO	Female subfertility	Defective cumulus matrix expansion.	(88, 89)
*Psen1*	Aspartic protease	Endoplasmic reticulum, Golgi, endosome, plasma membrane	KI (Leu166Pro)	Female infertility	Primordial follicles near the ovarian cortex and consisting largely of ovarian stromal elements.	(90)
*Adamts1*	Metalloprotease	Extracellular	KO	Female subfertility	Fewer numbers of mature follicles in ovary, thick and convoluted uterus.	(91, 92)
*Lonp*	Serine protease	Mitochondria	*Gdf9-cre* or *Zp3-cre*; *Lonp1* ^fl/fl^	Female infertility	Impaired follicular development, progressive oocyte death, ovarian reserve loss.	(93)
*Furin*	Serine protease	Golgi, endosome, plasma membrane, extracellular	*Gdf9-cre* or *Zp3-cre*; *Furin* ^fl/fl^	Female infertility	Arrest of early secondary follicles.	(94)
*Pappa*	Metalloprotease	Extracellular	KO	Female subfertility	Reduced litter size and reduced ovulatory capacity, probably because of decreased bioavailability of ovarian insulin-like growth factor.	(95)
*Astl*	Metalloprotease	Extracellular	KO	Female subfertility	No ZP2 cleavage after fertilization.	(96)
*Fetub*	Metalloprotease inhibitor	Extracellular	KO	Female infertility	Premature zona pellucida hardening.	(97)
*Serpinc1*	Serine protease inhibitor	Extracellular	KI (Arg48Cys)	Female subfertility	Thrombosis in placenta and penile vessels.	(98)
*Adam10*	Metalloprotease	Cell membrane	*Tie2-cre*; *Adam10* ^fl/fl^	Female subfertility	Impaired decidualization.	(99)
*Adamts18*	Metalloprotease	Extracellular	KO	Female infertility or subfertility	Fifty percent of mutant females are infertile because of vaginal obstruction due to either a dorsoventral vaginal septum or imperforate vagina.	(100)
*Plg*	Serine protease	Extracellular	KO	Female subfertility	Compromised female fertility.	(101, 102)
*Timp1*	Metalloprotease inhibitor	Extracellular	KO	Female subfertility	Reduction in reproductive lifespan.	(103)
*Pcsk2*	Serine protease	Extracellular	KO	Female subfertility	Details are uncertain.	(104)
*Espl1*	Cysteine protease	Nucleus	KI *Meox2^cre^*; *Espl1* ^+/S1121A^	Male infertility	Spermatogonia cell depletion.	(105)
			*Zp3-cre*; *Espl1* ^fl/fl^	Female infertility	Prevention of chiasmata resolution. Failure to extrude polar bodies in Meiosis I.	(106)
			*Meox2^cre^*; *Espl1* ^+/S1121A^	Female infertility	Primordial germ cell depletion by apoptosis during embryonic oogenesis.	(105, 107)
			*Zp3-cre*; KI (Ser1121Ala)	Female infertility	Failure in preimplantation development.	(108)
*Agtpbp1*	Metalloprotease	Intracellular	Spontaneous mutation, insertional mutation	Male infertility	Defective spermatogenesis.	(109–112)
				Female subfertility	Poor development of secondary follicles into antral follicles.	(113)
*Clpp*	Serine protease	Mitochondria	KO	Male infertility	Disrupted spermatogenesis at the spermatid stage.	(114)
				Female infertility	Ovarian follicular differentiation failure, premature reproductive aging.	(114)
*Npepps*	Metallo-aminopeptidase	Nucleus, cytosol	Gene trap	Male infertility	Lack of copulatory behavior, impaired spermatogenesis.	(115)
				Female infertility	Impaired formation of corpus luteum in pregnancy.	(116)
*Ggt1*	Protease	Plasma membrane	KO	Male infertility	Reduced testis and seminal vesicle size, reduced seminiferous tubule diameter.	(117)
				Female infertility	Hypogonadal, absence of antral follicles and corpora lutea and follicular degeneration.	(117)
*Immp2l*	Serine protease	Mitochondria	KO	Severe male subfertility	Erectile dysfunction.	(118)
				Female infertility	Defective folliculogenesis and ovulation.	(118)
*Adam17*	Metalloprotease	Extracellular	*Sox9-cre*; *Adam17* ^fl/fl^	Male subfertility	Details are uncertain.	(119)
				Female infertility	Details are uncertain.	(119)
***Mesocricetus auratus***						
*Acr*	Serine protease	Acrosome	KO	Male infertility	Sperm failure in zona pellucida penetration.	(120)
***R. norvegicus***						
*Adamts16*	Metalloprotease	Extracellular	KO	Male infertility	Cryptorchidism.	(121, 122)
***D. rerio***						
*adamts9*	Metalloprotease	Extracellular	KO	Female infertility	Ovary malformation.	(123)
***H. sapiens***						
*SPINK2*	Serine protease inhibitor	Extracellular	Spontaneous mutation	Male infertility	Azoospermia.	(59)
*GCNA*	Metalloprotease	Nucleus	Spontaneous mutation	Male infertility	Non-obstructive azoospermia and cryptoospermia.	(124, 125)
***A. thaliana***						
*A36*	Aspartic protease	Plasma membrane	T-DNA insertion	Decreased male transmission	Reduced pollen germination.	(126)
*A36*; *A39*	Aspartic protease	Plasma membrane	Double KO by T-DNA insertion	Severely compromised male transmission	Programmed cell death of microspores. Compromised micropylar guidance of pollen tubes.	(126)
*PCS1*	Aspartic protease	Endoplasmic reticulum	T-DNA insertion	Reduced male and female transmission	Degeneration of both male and female gametophytes.	(127)
*UND*	Aspartic protease	Mitochondria	siRNA and artificial microRNA	Partial male sterility	Apoptosis-like programmed cell death in tapetum and pollen.	(128)
*CEP1*	Cysteine protease	Vacuole, endoplasmic reticulum	T-DNA insertion	Male subfertility	Mutants exhibited aborted tapetal PCD and decreased pollen fertility with abnormal pollen exine.	(129)
*SPF1*; *SPF2*	SUMO-specific cysteine protease		Double KO by T-DNA insertion	Male and female sterility	Severe abnormalities in microgametogenesis, megagametogenesis, and embryo development.	(130)
***O. sativa***						
*OsAP65*	Aspartic protease	Vacuole	T-DNA insertion	Male sterility	No germination or elongation of mutant pollen.	(131)

## Unicellular Organisms

### Saccharomyces cerevisiae

*S. cerevisiae*, Baker’s yeast, is a model diploid unicellular organism. *S. cerevisiae* can stably exist as either a diploid or a haploid. When stressed, *S. cerevisiae* can undergo meiosis to produce four haploid spores. Haploid cells are capable of fusing with other haploid cells of the opposite mating type (an ‘a’ cell can only mate with an ‘α’ cell, and vice versa) to produce a stable diploid cell. a and α cells produce mating peptide pheromones a-factor and α-factor, respectively. Ste24p and Axl1p encoded by *ste24* and *alx1*, respectively, are metalloendopeptidases that process precursor peptide to produce mature mating a-factor pheromone (8, 9).

## Multicellular Organisms I: Invertebrates

The body of multicellular organisms consists of two types of cells with different lineages, i.e., germ cells and somatic cells. Germ cells produce gametes for fertilization, whereas somatic cells develop reproductive organs to support gametogenesis and fertilization by germ cells. Therefore, dysfunction of proteolysis in either cell lineage can result in fertility defects.

### Nematodes

*Caenorhabditis elegans* is androdioecious; i.e., it has two sexes, hermaphrodite and male, whereas *Ascaris suum* is dioecious, being either male or female. They develop two U-shaped gonads in which gametes are generated and fertilization occurs. Several proteases and inhibitors have been identified to regulate nematode reproductive processes.

Oogenesis and fertilization are affected when *cpi-2a*, encoding a cystatin-like cysteine protease inhibitor, is mutated (10). Nullification of *dss-1* encoding a 26S proteasome subunit provokes sterility because of deficient oogenesis (14). Knockdown of puromycin-sensitive aminopeptidase encoded by *pam-1* causes delayed oocyte maturation and subfertility (17). Deletion of *dpf-3* encoding a serine protease causes sterility because of impaired spermatogenesis (15). *gon-1* encoding a disintegrin-like and metalloproteinase domain with thrombospondin type 1 motif (ADAMTS) is necessary for morphogenesis of U-shaped gonads (11, 12). A mutant worm lacking *timp-1* encoding a tissue inhibitor of metalloproteinase also shows deficient gonadal development (13). A double mutant in which *sup-17* and *adm-4*, encoding nematode orthologs of mammalian membrane metalloproteases ADAM10 and ADAM17, respectively, are sterile because of aberrant spermathecal function (16).

Unlike mammalian flagellated sperm, nematode sperm are amoeboid cells. For successful fertilization, sperm must be activated prior to contacting an oocyte in both *C. elegans* and *A. suum*. This sperm activation is called spermiogenesis through which round immobile spermatids transform into motile, fertilization-competent spermatozoa. Mechanistically, spermiogenesis occurs by sensing extracellular signals and can be reproduced *in vitro* by exposing spermatids to proteases such as Pronase and proteinase K. A trypsin-like secreted protease encoded by *try-5* is expressed in the vas deferens and triggers activation of spermatids (18). *swm-1* encodes a secreted protein with a trypsin inhibitor-like domain, and *swm-1* mutant males are infertile because of ectopic premature activation of sperm (19). Like in *C. elegans*, activation of spermatozoa by exposure to extrinsic protease *in vitro* can also be seen in several insect species (132, 133). *spe-4* encoding﻿ a presenilin, an aspartyl protease with intramembrane proteolytic activity prevents spermatid activation because *spe-4* mutant males progress directly to functional spermatozoa without the need for an activation signal (134).

*gcna-1* encodes nuclear metalloprotease. *gcna-1* deletion causes genomic instability decreasing fertility in later generations (20). *T12E12.6* encodes intracellular metalloprotease whereas *zmp-2* encodes secreted metalloproteases. Knockdown of either of them results in reduced offspring production (17, 21).

### Insects

The reproductive system of *Drosophila melanogaster* is more complex compared with nematodes; it is composed of gonads, genital ducts, and accessory structures. Several proteases have been implicated in *D. melanogaster* spermatogenesis. In the *D. melanogaster* genome, there are five genes paralogous to *S. cerevisiae ste24* encoding a type I prenyl protease. Deletion of three tandemly arrayed *ste24* paralogs results in male fertility defects manifesting late in spermatogenesis (22).

All *Drosophila* spermatid nuclei descended from a primary spermatocyte remain connected to each other *via* an extensive network of cytoplasmic bridges. Spermatids should therefore be physically dissociated from each other by a process referred as individualization and a ubiquitin-proteasome system regulates this process. Males in which *Prosalpha6T* encoding a testis-specific proteasome core particle subunit was ablated are sterile because of defects in sperm individualization and nuclear maturation (23). *Duba* encodes a deubiquitylating enzyme and *Duba* null mutants are male sterile and display defects in spermatid individualization (24). The non-apoptotic function of caspases also contributes to individualization. DARK is a *Drosophila* homolog of mammalian caspase activator Apaf-1, whereas DRONC and DREDD are *Drosophila* apical caspases. Flies deficient in DARK or expressing a dominant-negative version of DRONC failed individualization (25, 135). *Dredd*-null flies also often show individualization defects (25).

In *D. melanogaster* sperm, mitochondrial derivatives run along the entire flagellum to provide structural rigidity for flagellar movement. Two mitochondrial derivatives (i.e., major and minor) differentiate and major one accumulates paracrystalline material by the end of spermatogenesis. S-Lap1-8, Sperm-Leucylaminopeptidase (S-Lap) family members are constituents of paracrystalline material. S-Lap mutants possess defects in paracrystalline material accumulation and abnormal structure of the elongated major mitochondrial derivatives and male sterility (28). *Htra2* encodes a mitochondrial serine protease. In one *Htra2*-null mutant line males are infertile because sperm are completely immotile (26), whereas spermatogenesis is defective in another *Htra2* mutant line (27).

Seminal fluid produced in the accessory gland includes proteases and protease inhibitors and is thought to contribute to fertilization in a post-mating manner. Seminase is a trypsin-like protease encoded by *Sems* and included in seminal fluid. When females mated with *Sems* knockdown males, they laid significantly fewer eggs (29). In cricket, *Allonemobius socius*, an ejaculate serine protease encoded by *ejac-sp* is expressed in male reproductive accessory glands. RNAi knockdown of *ejac-sp* resulted in a significant reduction of the male’s ability to induce a female to lay eggs (38). *Nep4*, a drosophila ortholog of mammalian *Mmel1*, encodes a metalloprotease expressed in male gonads (136). *Nep4* mutant males are infertile; mutant sperm are quickly discarded by females (30). When *Dcp-1* encoding a cysteine protease was ablated in their germline, the resulting females were infertile because of defective oogenesis (31).

Several proteases also of concern in *Drosophila* reproduction include *maternal haploid* or *mh* encodes the *Drosophila* homolog of SPRTN, a conserved metalloprotease essential for resolving DNA–protein cross-linked products. Paternal chromatids of *mh* mutants are unable to separate in the anaphase of the first embryonic mitosis and form a chromatin bridge. As a consequence, haploid nuclei of maternal origin rapidly separate from the damaged paternal chromosomes and haploid embryos develop but become lethal in a maternal effect manner (32, 33, 137). *Ance* encodes a putative homologue of mammalian angiotensin-converting enzyme (ACE). Compound heterozygote for two different *Ance* lethal alleles exhibit male sterility (34), but the molecular details are unknown. RNAi knockdown of *Slfc* encoding a secreted serine protease causes male infertility (35). When a membrane serine protease encoded by *ome* was mutated, males became subfertile (36). RNAi knockdown of a secreted metalloprotease encoded by *Mmp2* caused female subfertility because ovulation was blocked (37).

Several pest control attempts target reproduction-associated proteases. In pests *Spodoptera litura* and *Plutella xylostella*, targeted inactivation of serine protease genes *Osp* and *Ser2*, respectively, resulted in female and male infertility as also observed in silkworm moth *Bombyx mori* (39, 40). In other pests *Hyphantria cunea*, and *Bactrocera dorsalis*, RNAi knockdown of *Hcser2*, and *Bdcp-1* encoding serine protease and cysteine protease, respectively, also resulted in infertility (41, 42). Thus, proteases are potential targets for pest population control.

## Multicellular Organisms II: Vertebrates

Findings in vertebrates were obtained by genetic studies in rodents, fish, and human patients. Genes disrupted in these species include those encoding proteases, protease inhibitors, and non-catalytically active pseudo-proteases. Proteolysis-related factors are included in various aspects of male and female reproductive processes such as gamete production, gamete maturation, fertilization, post-fertilization events, and mating behavior.

### UPS in Gamete Production

For the fine-tuning of cellular processes, intracellular proteins are timely degraded by UPS. The proteasome localizes in the nucleus and cytoplasm where it degrades ubiquitylated proteins. Spermatoproteasome, a testis-specific proteasome, is one of the three tissue-specific proteasomes identified together with the immunoproteasome and the thymoproteasome in mammals (138). Deletion of *Psma8*, which encodes a testis-specific 20S proteasome component, leads to spermatogenesis arrest at the spermatocyte stage (43). *Psme3* encodes REGγ, a proteasome activator. *Psme3*-null males are subfertile with decreased sperm number and motility (44). This is probable because REGγ regulates p53-mediated transcription of *Plzf*, a transcription factor necessary for spermatogonial stem cell self-renewal and proliferation (139). *Psme4* encodes PA200 proteasome activator. *Psme4*-null males have reduced fertility due to defects in meiotic spermatocytes and post-meiotic spermatids (45). *Psme3*;*Psme4* double KO males were infertile; mutant sperm appeared morphologically normal but exhibited remarkable defects in motility and decreased proteasome activity (46).

Proteasome target proteins are ubiquitylated by E3 ubiquitin ligases which transfer the ubiquityl group from E2 ligase to the target protein. There are ∼600 E3 ligases encoded in the mammalian genome (140). The ubiquitin ligases, which are not proteases but included in ubiquitin-proteasome system-mediated protein degradation, indispensable for mammalian reproduction are listed in **Table 2**. Here only Huwe1 is mentioned as how E3 ligases function in reproductive processes. Huwe1 ubiquitylates histone H2AX, which is phosphorylated in response to DNA damage and is essential to the efficient recognition and repair of DNA double-strand breaks. Germline-specific *Huwe1* ablation increased histone H2AX level, elevated DNA damage response, and caused Sertoli cell only phenotype. Thus Huwe1 likely regulates the response to spontaneous DNA damage by UPS-mediated H2AX degradation to maintain cell survival (156).

**Table 2:** The ubiquitin ligases indispensable for mammalian reproduction.

Gene	Type	Gene-modified organism	Phenotype	Refs.
***D. melanogaster***				
*rae1*	E3 ligase component	*ms (2*)*Z5584* mutation	Male infertile, striking defects in primary spermatocyte nuclear integrity, meiotic chromosome condensation, segregation, and spindle morphology.	(141)
*parkin*	E3 ligase	P element insertion	Female infertility.	(142)
*cul3*	E3 ligase	EMS mutagenesis	Male infertility	(143)
***C. elegans***				
*mel-26*	E3 ligase	EMS mutagenesis	Germ cell depletion and sterility.	(144)
*skr-1*, *skr-2*	E3 ligase component	RNAi knockdown	Hermaphrodites are sterile. Arrested germline development in pachytene stage, expanded transition zone, and the presence of gaps in the gonad arm.	(145)
*vhl-1*	E3 ligase	RNAi knockdown	Reduced fertility.	(146)
***M. musculus***				
*Chfr*	E3 ligase	KO	30% of KO male were infertile.	(147)
*Cul4a*	E3 ligase component	KO	Male infertility phenotype resulted from a combination of decreased spermatozoa number, reduced sperm motility and defective acrosome formation.	(148, 149)
*Cul4b*	E3 ligase	*Vasa-cre*; *Cul4b* ^fl/Y^	Male infertility.	(150)
		*Cul4b* ^-/Y^	Male infertility.	(151)
*Dcaf17*	E3 ligase	KO	Male infertility due to abnormal sperm development.	(152)
*Dcaf8*	E3 ligase	KO	Pronounced sperm morphological abnormalities with typical bent head malformation.	(153)
*Dcun1d1*	E3 ligase component for neddylation	KO	Malformed spermatozoa with supernumerary and malpositioned centrioles.	(154)
*Fbxw7*	E3 ligase component	*Amh-cre*; *Fbxw7* ^fl/fl^	Impaired testis development, which is characterized by age-dependent tubular atrophy, excessive germ cell loss, and spermatogenic arrest, and the mutant males were infertile at 7 months old	(155)
*Huwe1*	E3 ligase	*Ddx4-cre*; *Huwe1* ^fl/Y^	Male infertile, Sertoli cell only phenotype. Increased level of histone H2AX and an elevated DNA damage response.	(156)
	E3 ligase	*Stra8-cre*; *Huwe1* ^fl/Y^	Male infertile, spermatogenesis arrest. Accumulation of DNA damage response protein γH2AX.	(157)
	E3 ligase	*Zp3-cre*; *Huwe1* ^fl/fl^	Oocyte death and female infertility.	(158)
*Mdm2*	E3 ligase	*Pgr-cre*; *Mdm2* ^fl/fl^	Female infertility. Impaired oocyte maturation, ovulation, and fertilization.	(159)
		*Gdf9-cre*; *Mdm2* ^fl/fl^	Female infertility. Complete lack of follicular structures resembling human premature ovarian failure.	(160)
		*Zp3-cre*; *Mdm2* ^fl/fl^	Female infertility.	(160)
		*Amh-cre*; *Mdm2* ^fl/fl^	Male infertile. degenerated testes with no organized seminiferous tubules and a complete loss of differentiated germ cells.	(161)
*Mgrn1*	E3 ligase	Spontaneous	Male infertility.	(162)
*Phf7*	E3 ligase	KO	Male infertility due to impaired protamine replacement in elongated spermatids.	(163)
*Rnf20*	E3 ligase	*Stra8-cre*; *Rnf20* ^fl/fl^	Male infertility because of arrested spermatogenesis at the pachytene stage.	(164)
*Rnf216*	E3 ligase	KO	Disrupted spermatogenesis and male infertility.	(165)
*Rnf8*	E3 ligase	KO	Male infertility.	(166)
		Gene trap	Male infertility.	(167)
*Siah1a*	E3 ligase	KO	Female subfertility and male infertility. Interrupted spermatogenesis because of impaired progression past meiotic metaphase I.	(168)
*Spop*	E3 ligase	*Pgr-cre*; *Spop* ^fl/fl^	Female infertility because of impaired uterine decidualization.	(169)
*Syvn1 (Hrd1)*	E3 ligase	*Alb-cre*; *Hrd1* ^fl/fl^	Female infertility.	(170)
*Trim37*	E3 ligase	KO	Male and female infertility.	(171)
*Trim71*	E3 ligase	*Nanos3-cre*; *Trim71* ^fl/–^	Male infertility because of Sertoli cell-only phenotype.	(172)
*Ubr2*	E3 ligase	KO	Male infertility caused by arrested spermatogenesis at meiotic prophase I.	(173)
*Uhrf1*	E3 ligase	*Stra8-cre*; *Uhrf1* ^fl/fl^	Failure of meiosis and male infertility.	(174)
		*Zp3-cre*; *Uhrf1* ^fl/fl^	Female infertility.	(175)
*Rad6b*	E2 ligase	KO	Male infertility because of the loss of spermatogenesis	(166)
*Ube2i*	E2 ligase	*Gdf9-icre*; *Ube2i* ^fl/fl^	Female infertility with major defects in stability of the primordial follicle pool, ovarian folliculogenesis, ovulation and meiosis.	(176)
*Ube2j1*	E2 ligase	KO	Male infertility because of deficient spermatogenesis.	(177)
*Ube2q1*	E2 ligase	KO	Reduced female fertility. Altered estrus cycle, abnormal sexual behavior and reduced offspring care, and significantly increased embryonic lethality in the uterus of mutant females.	(178)
***H. sapiens***				
*RNF220*	E3 ligase	Spontaneous mutation	Small-headed sperm.	(179)
***A. thaliana***				
*PUB4*	E3 ligase	T-DNA insertion	Male sterility.	(180)
*SAP*	E3 ligase component	Two-element Enhancer-Inhibitor transposon system	Male and female sterility. Severe aberrations in inflorescence and flower and ovule development. Carpelloid sepals, short and narrow or absent petals, and degenerated anthers.	(181)
*SIZ1*	SUMO E3 ligase	T-DNA insertion	Arrest of funicular and micropylar pollen tube guidance.	(182)
*MMS21*	SUMO E3 ligase	T-DNA insertion	Severely reduced fertility, deficient gametogenesis.	(183)
***O. sativa***				
*SIZ1*	SUMO E3 ligase	T-DNA insertion	Spikelet sterility caused by defective anther dehiscence.	(184)

Cullin-RING E3 ubiquitin ligases are known to be reversibly neddylated, i.e., conjugated with NEDD8, a ubiquitin-like protein. By conjugation with NEDD8, cullin-RING E3 ligases increase their stability and ligase activity. The constitutive photomorphogenic-9 signalosome (CSN) deneddylates cullin-RING E3 ligases by cleaving the isopeptide bond of neddylated lysine to regulate the cellular ubiquitylation status. COPS5 is the fifth component of the CSN and abundant in mouse testis (185). *Cops5-*null males were infertile because of significant reduction of sperm number caused by premeiotic apoptosis of germ cells (47).

Ubiquitylated proteins can be deubiquitylated by deubiquitylating enzymes such as ubiquitin-specific proteases (USPs), cysteine endopeptidases encoded by *Usp* genes, thereby expression levels and activity of target proteins are regulated. USP1 deubiquitylates FANCD2 which is included in the repair of DNA crosslinks. *Usp*1 null males were infertile and the seminiferous tubules were markedly atrophic and mostly devoid of spermatogenic cells in the mutant testis. *Usp2*-null males possessed severely reduced fertility and the mutant sperm were defective in sperm motility and egg fertilizing ability *in vitro* (48). Germ cell-specific ablation of *Usp9x* using *Vasa-cre* possessed spermatogenic cell apoptosis at the early spermatocyte stage and resulted in complete infertility (49). *Usp26* is an X-linked gene exclusively expressed in testis (186). *Usp26* -null males are subfertile because of reduced number of haploid cells in testis (50, 51). *Usp*1-null female mice showed reduced fertility probably because of a reduced number of oocytes in ovaries (52). Thus, UPS is critically important for germ cell production in both sexes.

### Non-Proteasomal Intracellular and Extracellular Proteolysis Factors in Sperm Production

Intracellular and extracellular proteolysis factors critically function in spermatogenesis. Cleavage of specific peptide bonds also contributes to spermatogenesis. *Apaf1* encodes a caspase activator, and *Apaf1*-null males are infertile because of degeneration of spermatogonia, which results in the absence of sperm (53). *Agbl5* encodes an intracellular metalloprotease. *Agbl5*-null males are infertile because of defective spermatogenesis (54, 55). A cytosolic carboxypeptidase 1, another metalloprotease encoded by *Agtpbp1* deglutamylates polyglutamylated proteins. *Agtpbp1* mutant mice known as *Purkinje cell degeneration* (*pcd*) possess male infertility (109–112) because of defective spermatogenesis (110). A germ cell nuclear antigen encoded by *Gcna* contains a metalloprotease domain. *Gcna*-null males are nearly devoid of sperm and infertile (56). In human, *GCNA* spontaneous mutations were identified in spermatogenic failure patients (124, 125).

Separin, a caspase-like cysteine protease encoded by *Espl1*, plays a central role in chromosome segregation by cleaving the SCC1/RAD21 subunit of the cohesin complex (187–189). A point mutation in *Espl1* which substitutes inhibitory phosphorylation site Ser^1121^ to Ala depletes spermatogonia because of chromosome misalignment during proliferation of the postmigratory primordial germ cells and following mitotic arrest, aneuploidy, and cell death (105). Threonine aspartase 1 (TASP1) is an intracellular endopeptidase that cleaves after distinct aspartate residues of the conserved IXQL(V)D/G motif (190). TASP1 cleaves general transcription factor TFIIAα−β to enable testis-specific transcription; *Tasp1*-null male mice were unable to activate spermatogenic gene activation, which lead to the release of immature germ cells and infertility (57). A serine protease ClpP is located in the mitochondrial matrix and participates in mitochondrial protein quality control by degrading misfolded or damaged proteins. In *Clpp*-null mutants spermatogenesis was disrupted by the spermatid stage (114). *Tysnd1* encodes a serine protease that processes peroxisomal leader peptides. *Tysnd1-*null mutant males possess globozoospermia and their spermatozoa lack the acrosomal cap (58). *Spink2* encodes a Kazal-type serine protease inhibitor abundantly expressed in testis and epididymis (191). *Spink2*-null males had azoospermia, and a homozygous splice mutation of *SPINK2* was found in infertile men (59). Ablation of *Serpina5* encoding another serine protease inhibitor also results in an abnormality in sperm production in the testis (60).

Puromycin-sensitive aminopeptidase encoded by *Npepps* is also an intracellular protease. It appears to contribute indirectly to spermatogenesis. *Npepps*-null testes and seminal vesicles were significantly reduced in weight, spermatogenesis was impaired, and copulatory behavior was lacking. It is suggested that the defects in the testes likely arises from dysfunction of Sertoli cells, whereas the lack of copulatory behavior results from defects in the brain (115).

A null mutation of *Adamts2* encoding secreted metalloproteinase caused male infertility (61). Decreased spermatogenesis was observed but copulatory behavior and/or copulatory plug formation may also be impaired because a copulatory plug was never observed (61).

### Proteolysis Factors Associated With Sperm Function

#### Acrosomal Function

The acrosome is a Golgi-derived sperm head organelle in which many digestive enzymes such as proteases and hyaluronidases are included to penetrate egg surroundings. Acrosin is a serine protease and a major component of the acrosome. Although acrosin-deficient male mice are fertile (62, 63), disruption of hamster acrosin resulted in complete male infertility (120). *In vitro*, mutant hamster spermatozoa attached to the zona pellucida, but failed to penetrate it (120), suggesting that acrosomal function can be attributed to specific factors in a species-specific manner.

Proprotein convertases convert inactive precursor proteins into their mature and active forms. PCSK4 is a member of proprotein convertases expressed on the sperm surface overlying the acrosome (64). *Pcsk4*-null males showed impaired fertility (64, 65) and mutant sperm exhibited accelerated capacitation, precocious acrosome reaction, reduced binding to egg zona pellucida (64). Acrosome formation during spermatogenesis was also abnormal (192).

#### Sperm Maturation

A group of genes encoding proteases, enzymatically inactive pseudoproteases, and protease inhibitors is apparently associated with the same physiological function, i.e., maturation of sperm conferring abilities to migrate into female oviduct and bind with zona pellucida. Ablation of *Tmprss12* (66), *Prss55* (67, 68), *Tryx5* (69), *Prss37* (70), *Ace* (71), *Adam1a* (72), *Adam2* (73), *Adam3* (74, 75), and *Adam6* (76) results in deficient sperm migration into the oviduct and binding to the zona pellucida of eggs. Among them, *Adam1a*, *Adam2*, *Adam3*, *Adam6*, and *Prss37* encode catalytically inactive pseudoproteases. A disintegrin and metallopeptidase domain (ADAM) 3, a catalytically inactive transmembrane pseudoprotease appears to be central to a molecular mechanism that governs sperm migratory and adhesion abilities, because ADAM3 expression is a prerequisite for sperm to acquire these abilities (193).

ADAM3 is expressed as a precursor and the processed into mature form as spermatozoa mature in epididymis (194). Similarly, enzymatically inactive pseudoproteases ADAM2 and ADAM6 are processed during sperm maturation in epididymis (195, 196). Therefore, they are rather substrates for other proteases. Ablation of ADAM2 or ADAM6 also results in significant decrease or loss of ADAM3 from epididymal sperm (74, 76) indicating the involvement of both ADAM2 and ADAM6 in ADAM3 expression. PRSS37 supports ADAM3 precursor translocation to the sperm cell surface by collaborating with PDILT, a testis-specific protein disulfide isomerase indispensable for ADAM3 surface expression (197, 198). TMPRSS12, PRSS55, and TRYX5, all of which are serine proteases and retain catalytic triad residues, are necessary for the production or stable localization of processed ADAM3 on the cell surface of epididymal spermatozoa (66–69), although it remains uncertain whether these proteases directly cleave ADAM3.

Cystatins are secreted cysteine proteinase inhibitors. Cystatin genes *Cst8*, *9*, *11*, *12*, *13*, *dc1*, *dc2*, and *l1* are clustered on mouse chromosome 2 and expressed in both testis and epididymis. Their simultaneous ablation resulted in the loss of ADAM3 from epididymal sperm and deficient sperm migration into the oviduct (77), implying the importance of regulated proteolysis in sperm maturation. Ovochymase 2 (OVCH2) is a chymotrypsin-like serine protease. OVCH2 is specifically expressed in the caput epididymis under the regulation of lumicrine signaling, in which testis-derived secreted protein NELL2 transiting through the luminal space acts on the epididymal epithelium by binding to its receptor ROS1 tyrosine kinase to differentiate (78). Ablation of *Ovch2* results in abnormal sperm ADAM3 processing and deficient sperm migration into the oviduct (78). Thus, regulated proteolysis on or outside spermatozoa apparently modulates sperm maturation.

NL1 encoded by *Mmel1* is a zinc metallopeptidase expressed in testis. NL1 is expressed as a type II transmembrane protein but released as a soluble form. *Mmel1*-null mice show normal spermatogenesis but reduced egg fertilization, suggesting the role of NL1 in sperm maturation (79). It remains, however, uncertain whether NL1 is included in ADAM3-mediated sperm maturation. Testisin encoded by *Prss21* is a GPI-anchored serine protease. *Prss21* KO males are subfertile because mutant spermatozoa possessed decreased motility, angulated and curled tails, and fragile necks (80). In another *Prss21* mutant line *in vitro* sperm binding to egg zona pellucida, acrosome reaction, and fertility were decreased (81).

### Other Proteolytic Factors Associated With Male Reproduction

Several cell surface and extracellular proteases and inhibitors seem to regulate male fertility in more indirect manners. *Adamts16* homozygous mutant rat males resulted in cryptorchidism and male sterility (121). The mutant testis undescended during development because of the failure of gubernacular migration (122). γ-glutamyltranspeptidase 1 (GGT1) is a type II transmembrane protein which cleaves γ-glutamyl bond of extracellular glutathione (γ-Glu-Cys-Gly), glutathione conjugates, and other γ-glutamyl compounds. The resulting cysteinyl-glycine is further cleaved by dipeptidase into free amino acids. *Ggt1-*null males are infertile because of decreased epididymal sperm number and failure in copulatory plug formation (117). Although *Ggt1-*null testis was small, spermatogenesis inside seminiferous tubules appeared normal and seminal vesicles were hypoplastic. As *N*-acetylcysteine-fed mutant mice were fertile, the observed infertility is a consequence of cysteine deficiency (117),. Carboxypeptidase E (CPE) is a metallo-carboxypeptidase and functions as a prohormone processing exopeptidase. *Cpe^fat^* ^/^ *^fat^* males are infertile and deficient in Pro-gonadotropin-releasing hormone processing in the hypothalamus (82). ADAM24 is a metalloproteinase localized on the mature sperm surface. *Adam24*-null males are subfertile and polyspermic fertilization increased *in vitro* and *in vivo*, suggesting a physiological role of ADAM24 for prevention of polyspermy (83). ADAM7 is a membrane-anchored protein with a catalytically-inactive metalloproteinase domain abundantly expressed in the epididymis (199). *Adam7* ablation resulted in a modest reduction of male fertility; impaired epididymal morphology and integrity may affect sperm maturation (84).

Cystatin C encoded by *Cst3* is a cysteine protease inhibitor abundantly expressed in testis and epididymis. Substitution of Leu^68^ to Gln is an amyloid-forming mutation found in a hereditary form of cystatin C amyloid angiopathy. Heterozygous male mice were infertile and increased levels of amyloid was observed in the epididymal fluid (85). Nonpathological function of amyloid during epididymal sperm maturation is also suggested (200).

*Immp2l* encodes an inner mitochondrial membrane peptidase 2-like. *Immp2l*-null homozygous males were severely subfertile because of erectile dysfunction (118). Tumor necrosis factor-α (TNFα) converting enzyme encoded by *Adam17* is involved in the proteolytic release of the ectodomain of diverse cell surface proteins. Conditional ablation of *Adam17* with *Sox9-cre* severely impaired male fertility but the details are uncertain (119).

*Serpine2* encodes protease nexin-1, a serine protease inhibitor expressed in seminal fluid. *Serpine2*-null males possessed reduced fertility because of impaired semen coagulation and copulatory plug formation (86).

### Proteolytic Factors in Ovary and Follicle Development

Both intracellular and extracellular proteolytic factors are included in ovary and follicle development. Conditional ablation of separase under the control of *Zp3-cre* hindered extrusion of the first polar body and caused female sterility (106). Introduction of a Ser^1121^ to Ala deregulatory mutation into separase led to primordial germ cell apoptosis during embryonic oogenesis (107). Ablation of cytosolic carboxypeptidase 1 encoded by *Agtpbp1* results in female subfertility because secondary follicles poorly develop into antral follicles (113). Oocyte-specific ablation of nuclear cysteine protease separase causes female infertility because mutant oocytes are able neither to extrude polar bodies in meiosis I nor to resolve chiasmata (106).

A deregulatory mutation into separin encoded by *Espl1* at early embryonic period caused primordial germ cell depletion by apoptosis during embryonic oogenesis, which led to female infertility (105, 107). The introduction of the same mutation at later oocyte development by using *Zp3-cre* also resulted in female infertility but because of failure in preimplantation development (108).

Matriptase encoded by *Tmprss6* is a type II transmembrane serine protease which functions in iron homeostasis by cleaving cell surface proteins associated with iron absorption. *Tmprss6-*null females possessed marked retardation in ovarian maturation (87), probably because of severe decrease in plasma iron levels. The defective ovarian follicle development and female infertility can be mimicked by a low iron diet (201).

The inter-α-trypsin inhibitor (IαI) family are abundantly found in body fluids including blood plasma and urine and possess inhibitory activity for serine proteases. They are composed of bikunin, a proteoglycan with a single chondroitin sulfate chain, and heavy chains covalently bound to chondroitin sulfate chain of bikunin. IαI family members are able to transfer their heavy chains from IαI to hyaluronan in the presence of tumor necrosis factor-stimulated gene-6. This reaction results in the modified hyaluronan covalently linked heavy chain and is necessary for hyaluronan-rich cumulus matrix expansion. When the bikunin-coding region was deleted from *Ambp* gene, the resulting homozygous females ovulate oocytes deficient in hyaluronan-rich cumulus matrix expansion, leading to female infertility (88, 89).

γ-secretase is an endoprotease complex that catalyzes the intramembrane cleavage of integral membrane proteins. *Psen1* encodes presenillin-1, a catalytic subunit of γ-secretase. Female mice homozygous with ﻿a Leu^166^ to Pro mutation, an aggressive mutation found in familial Alzheimer’s disease patients, are infertile and their ovaries consisted largely of stromal elements with primordial follicles near the cortex (90).

ADAMTS1 is a secreted metalloproteinase expressed in the granulosa cell layer of mature follicles in the ovary (91). *Adamts1*-null females possessed lower numbers of mature follicles in the ovary and a thick and convoluted uterus (92). In another mutant mouse line, ovulation in null females was impaired because mature oocytes remained trapped in ovarian follicles (91). In zebrafish, *adamts9*-null females possess ovarian malformation and are unable to ovulate (123).

*Lonp* encodes a mitochondrial serine protease. Oocyte-specific *Lonp* ablation by *Gdf9-cre* or *Zp3-cre*; *Lonp1* ^fl/fl^ results in female infertility because of impaired follicular development, progressive oocyte death, ovarian reserve loss (93). *Furin* encodes a transmembrane serine protease localized in Golgi appratus, endosome, plasma membrane; it is necessary for mature protein release by cleaving at RX(K/R)R consensus motif. Conditional ablation of *Furin* by *Gdf9-cre* or *Zp3-cre*; *Furin* ^fl/fl^ result in female infertility because of the arrested oogenesis at early secondary follicles (94). *Pappa* encodes an extracellular metalloprotease. *Pappa* KO females decreased their litter size and ovulatory capacity, probably because of decreased bioavailability of ovarian insulin-like growth factor (95).

Loss of GGT1 causes infertility in not only males but females. In the *Ggt1*-null females, antral follicles and corpora lutea were absent and follicles degenerated due to the reduced intracellular cysteine levels (117).

Mitochondrial proteases also affect ovarian follicle development. Ablation of *Clpp* encoding mitochondrial matrix ClpP protease caused relatively small ovaries in which follicular differentiation was impaired probably because of the reduction of the granulosa cell layers (114). When the inner mitochondrial membrane peptidase 2-like encoded by *Immp2l* was ablated, the resulting mutant females were deficient in folliculogenesis and ovulation and infertile, probably because of low availability of nitric oxide caused by mitochondrial dysfunction (118).

### Proteolytic Factors in Post-Fertilization Events of Female Reproduction

Several proteolysis-associated secreted proteins contribute to post-fertilization events including the hardening of the egg-surrounding zona pellucida. Ovastacin encoded by *Astl* is a secreted metalloendopeptidase deposited in cortical granules of oocytes. Ovastatin is secreted into the extracellular space in response to egg activation triggered by fertilization. In *Astl*-null eggs, ZP2 cleavage necessary for zona pellucida hardening and the postfertilization block to polyspermy did not occur after fertilization (96). Fetuin is a cystatin family protease inhibitor abundantly expressed in blood plasma. Fetuin-B prevents premature ZP hardening probably by inhibiting ovastacin derived from spontaneous cortical granule release, as fetuin-B inhibited ovastacin protease activity *in vitro* and *Fetub*-deficient oocytes undergo premature zona pellucida hardening (97).

Antithrombin encoded by *Serpinc1* inhibits thrombin and some other coagulation factors by binding heparin and heparan sulfate. When an Arg^48^ to Cys mutation, which corresponds to human thrombosis mutation, was introduced into mice, the resulting homozygous females had decreased their litter size, probably because thrombosis occurred in placenta (98).

*Adam10* encodes a membrane metalloprotease. Conditional ablation of vascular *Adam10* by *Tie2-Cre*; *Adam10* ^fl/fl^ causes impaired decidualization and female subfertility (99). *Adamts18* encodes a member of secreted metalloprotease ADAMTS. *Adamts18*-null females suffer from vaginal obstruction, due to either a dorsoventral vaginal septum or imperforate vagina and infertility or subfertility (100).

### Other Proteolytic Factors in Female Reproduction

Several proteolysis-associated factors regulate female reproduction in a more indirect manner. *Npepps*-null females lacking a puromycin-sensitive aminopeptidase impairs corpus luteum formation and are infertile, probably because of disruption of the hypothalamic-pituitary axis (116). Plasmin is a secreted serine protease generated from plasminogen through activation by tissue-type or urokinase-type plasminogen activators. The fertility of plasmin-deficient *Plg*-null female mice appeared to be compromised (101, 102). It seems not to be the consequence of the impaired proteolytic process essential for ovulation, as plasminogen-deficient mice had normal ovulation efficiency (202). *Timp1* encodes a tissue inhibitor of metalloproteinases 1, an inhibitor for matrix metalloproteinases. *Timp1* mutation reduced the reproductive lifespan of female but not male mice (103). When *Pcsk2* encoding neuroendocrine convertase 2 was ablated, the number of consecutive litters from mutant female mice was small and *Pcsk2*-null female mice sometimes gave birth to dead pups (104) for uncertain reason. Conditional ablation of TNFα converting enzyme by *Sox9-cre*; *Adam17* ^fl/fl^ resulted in female infertility but details are uncertain (119).

## Fertility-Associated Proteases in Plants

Several aspartic proteases are associated with pollen development and function. In *Arabidopsis thaliana*, A36 and A39 are GPI-anchored putative aspartic proteases predominantly expressed in pollen and the pollen tube. In *a36*; *a39* double mutant, pollen grains underwent apoptosis-like programmed cell death and the pollen tube compromised micropylar guidance (126). *UND* encodes a secreted aspartic protease UNDEAD, and its silencing using small interfering RNA caused premature tapetal and pollen programmed cell death (128).

In *Oryza sativa*, *OsAP65* encodes an aspartic protease localized in the pre-vacuolar compartment. T-DNA-inserted *OsAP65* mutant alleles could not be transmitted through the male gamete; the mutant pollen matured normally, but did not germinate or elongate, indicating its essentiality in pollen germination and tube growth (131). *PCS1* encodes an aspartic protease and its loss-of-function mutation caused degenerated male and female gametophytes (127).

A cysteine protease also contributes to pollen development; when a papain-like vacuolar cysteine protease encoded by *CEP1* was ablated, the resulting mutants are male subfertile because of aborted tapetal programmed cell death and decreased pollen fertility with abnormal pollen exine (129).

Some aspect of *A. thaliana* reproduction includes Small Ubiquitin-related Modifier (SUMO). SPF1 and SPF2 are cysteine proteases and function in desumoylation of sumoylated proteins. *spf1*; *spf2* double mutants exhibit severe abnormalities in microgametogenesis, megagametogenesis, and embryo development (130). There are SUMO-E3 ligases involved in gametophyte development (182, 183) in *A. thaliana* and in anther dehiscence in *O. sativa* (184).

## Conclusion and Perspective

By a comprehensive survey, it has been demonstrated that proteolysis regulates reproduction in various species including yeast, insects, nematodes, vertebrates, and plants. Regulation of reproduction by proteolysis already exist in unicellular yeast. In multicellular organisms, proteolysis regulates the formation and function of gametes derived from germ cells as well as the development and function of reproductive organs by somatic cells, thereby securing successful reproduction. In these cell lineages, both limited proteolysis and degrative proteolysis by ubiquitin-proteasome system play critical roles.

One of intriguing paradigms emerging in this review is that many sperm surface and extracellular proteases, pseudoproteases, and inhibitors are included in the acquisition of mammalian sperm conferring abilities to migrate into the oviduct and to bind to the zona pellucida of eggs. As spermatozoa are transcriptionally and translationally silent, post-translational modification mechanisms such as proteolysis may largely contribute to sperm maturation.

Many compounds have been designed to inhibit the enzymatic activity of proteases. Clinically, there have been numerous successes including angiotensin-converting enzyme inhibitors for cardiovascular disorders (203), thrombin inhibitors for thromboembolism and bleeding disorders (204, 205), and HIV protease inhibitors in the treatment of HIV and AIDS (206), among others (207, 208). In addition, enzymatically active proteases could also be good druggable targets for contraceptives.

Genome editing techniques developed in recent years will identify fertility-associated proteolytic factors further. In addition to identifying novel factors, more intense studies on the molecular basis of proteolysis including the identification of substrates will clarify how proteolytic events govern reproduction. It will also clarify the physiological significance of molecular events governed by proteolysis in reproduction.

## Author Contributions

DK and MI wrote the manuscript. All authors contributed to the article and approved the submitted version.

## Funding

This work was supported in part by Ministry of Education, Culture, Sports, Science and Technology (MEXT)/Japan Society for the Promotion of Science (JSPS) KAKENHI grants (JP21H00231 to D.K. and JP21H05033 to MI), Japan Science and Technology Agency (21460710 to D.K. and 21467777 to MI), National Institutes of Health (R01HD088412 and P01HD087157 to MI), and the Bill & Melinda Gates Foundation (Grant INV-001902 to MI). Under the grant conditions of the Foundation, a Creative Commons Attribution 4.0 Generic License has already been assigned to the Author Accepted Manuscript version that might arise from this submission.

## Conflict of Interest

The authors declare that the research was conducted in the absence of any commercial or financial relationships that could be construed as a potential conflict of interest.

## Publisher’s Note

All claims expressed in this article are solely those of the authors and do not necessarily represent those of their affiliated organizations, or those of the publisher, the editors and the reviewers. Any product that may be evaluated in this article, or claim that may be made by its manufacturer, is not guaranteed or endorsed by the publisher.

## Abbreviations

ACE, angiotensin converting enzyme; ADAM, a disintegrin-like and metalloproteinase domain; ADAMTS, a disintegrin-like and metalloproteinase domain with thrombospondin type 1 motif; CSN, constitutive photomorphogenic-9 signalosome; EMS, ethylmethane-sulfonate; GGT, glutamyltranspeptidase; IαI, inter-α-trypsin inhibitor; KI, knock-in; KO, knockout; OVCH2, ovochymase 2; S-Lap, sperm-Leucylaminopeptidase; SUMO, small ubiquitin-related modifier; TASP1, threonine aspartase 1; TMP,trimethylpsoralen; TNFα, tumor necrosis factor-α; UPS, ubiquitin-proteasome system; USP, ubiquitin-specific protease.
